# Heavy Doping by Bromine to Improve the Thermoelectric Properties of n‐type Polycrystalline SnSe

**DOI:** 10.1002/advs.201800598

**Published:** 2018-07-31

**Authors:** Shan Li, Yumei Wang, Chen Chen, Xiaofang Li, Wenhua Xue, Xinyu Wang, Zongwei Zhang, Feng Cao, Jiehe Sui, Xingjun Liu, Qian Zhang

**Affiliations:** ^1^ Department of Materials Science and Engineering Harbin Institute of Technology Shenzhen Guangdong 518055 P. R. China; ^2^ Beijing National Laboratory for Condensed Matter Physics Institute of Physics Chinese Academy of Science Beijing 100190 P. R. China; ^3^ School of Science Harbin Institute of Technology Shenzhen Guangdong 518055 P. R. China; ^4^ State Key Laboratory of Advanced Welding and Joining Harbin Institute of Technology Harbin Heilongjiang 150001 P. R. China

**Keywords:** carrier concentration, highly doping, n‐type, thermoelectric

## Abstract

Single crystal tin selenide (SnSe) has attracted much attention for its excellent thermoelectric performance. However, polycrystalline SnSe exhibits unsatisfactory figure‐of‐merit due to the inferior electrical properties, especially for n‐type SnSe. In this work, a high concentration of Br doping (6–12 atm%) on the Se site effectively increases the Hall carrier concentration from 1.6 × 10^17^ cm^−3^ (p‐type) in undoped SnSe to 1.3 × 10^19^ cm^−3^ (n‐type) in Br‐doped SnSe_0.88_Br_0.12_, leading to an increased electrical conductivity close to that of a single crystal. Combined with the decreased lattice thermal conductivity due to the enhanced phonon scattering by composition fluctuation and dislocations, a peak *ZT* of ≈1.3 at 773 K, together with the enhanced average *ZT* is obtained in SnSe_0.9_Br_0.1_ along the hot pressing direction.

## 1 Introduction

The global problem of energy crisis and environmental issues impels the development of sustainable and clean energy technology. Thermoelectric (TE) materials can directly convert heat into electricity without moving parts and gaseous emission, having aroused wide attention for a global sustainable energy solution. The conversion efficiency of TE device is characterized by the dimensionless figure of merit *ZT* = *S* ^2^ *σT*/(κ_e_+κ_L_), where *S* is the Seebeck coefficient, σ the electrical conductivity, *T* the absolute temperature, κ_L_ the lattice thermal conductivity, and κ_e_ the electronic thermal conductivity.^1^, ^2^ Numerous approaches have been explored to enhance TE performance, including increased power factor (*S* ^2^σ) by band engineering (resonant doping, band convergence, and band flattening, etc.)^3^, ^4^, ^5^ and decreased thermal conductivity by defect engineering,^6^ and nanoengineering.^7^, ^8^, ^9^ An alternative way to achieve high *ZT* values is to seek new classes of TE materials with intrinsically low thermal conductivity, such as clathrates, skutterudites, sulfur‐based compounds, Ag_9_GaSe_6_, and SnSe *etc*.^10^, ^11^, ^12^, ^13^, ^14^

SnSe was well studied in solar cells and electronic memory applications, but ignored as the TE materials due to the large room temperature electrical resistivity (10^1^–10^5^ Ω cm).^15^, ^16^ However, a promising high *ZT* ≈2.6 at 923 K was obtained along the crystallographic *b* axis in single crystal SnSe due to the intrinsic ultralow thermal conductivity induced by strongly anharmonic bonding.^17^ Although there are some controversy regarding the actual thermal conductivity of SnSe single crystals, this enhancement is also attributed to the decreased electrical resistivity in single crystals (0.1 Ω cm) with higher Hall mobility, which is subsequently pursued in the polycrystalline SnSe. Since the electrical resistivity is in inverse proportion to the carrier concentration and mobility, the easiest way is to dope in polycrystalline SnSe for higher carrier concentration, considering the inevitably defects in polycrystals. Relatively high electrical conductivity and *ZT* values have been achieved in p‐type polycrystalline SnSe doped with Ag, Na, and K, etc.^18^, ^19^, ^20^, ^21^ Specifically, multiple valence bands have been activated by doping with Na for the further enhanced Seebeck coefficient and TE performance in SnSe. However, since intrinsic Sn vacancy shows p‐type conducting behavior, it is difficult to convert SnSe to n‐type conductor, much more difficult to activate the heavy conduction band^22^ if compared with p‐type SnSe. I,^23^ BiCl_3_,^24^ Bi,^25^ and Br^26^ have been proved capable of changing the Hall coefficient from positive to negative. A decent *ZT* of ≈0.8 at about 773 K was obtained in SnSe_0.96_I_0.04_ with room temperature electron carrier concentration ≈2.0 × 10^17^ cm^−3^.^23^ And an improved *ZT* value of ≈1.1 at 773 K was achieved in SnSe_0.97_Br_0.03_ with room temperature electron carrier concentration up to ≈1.83 × 10^18^ cm^−3^.^26^ To be noted that this carrier concentration is still too low and the electrical conductivity of this Br‐doped polycrystalline SnSe is still much lower than that of single crystal.

In this work, heavy (6–12 atm%) Br‐doped n‐type polycrystalline SnSe_1–_ *_x_*Br*_x_* was prepared by melting and hot pressing. The Hall carrier concentration increased dramatically from 1.6 × 10^17^ cm^−3^ (p‐type) in undoped SnSe to 1.3 × 10^19^ cm^−3^ (n‐type) in Br‐doped SnSe_0.88_Br_0.12_, leading to an increased electrical conductivity close to that of single crystal SnSe. Combined with a lowered lattice thermal conductivity at high temperature by effective defect scattering and suppression of the bipolar effect, both the average *ZT* and the highest *ZT* were improved. A highest *ZT* value of ≈1.3 was achieved at 773 K for SnSe_0.9_Br_0.1_ along the hot pressing direction.

## 2 Results and Discussion

**Figure 1:**
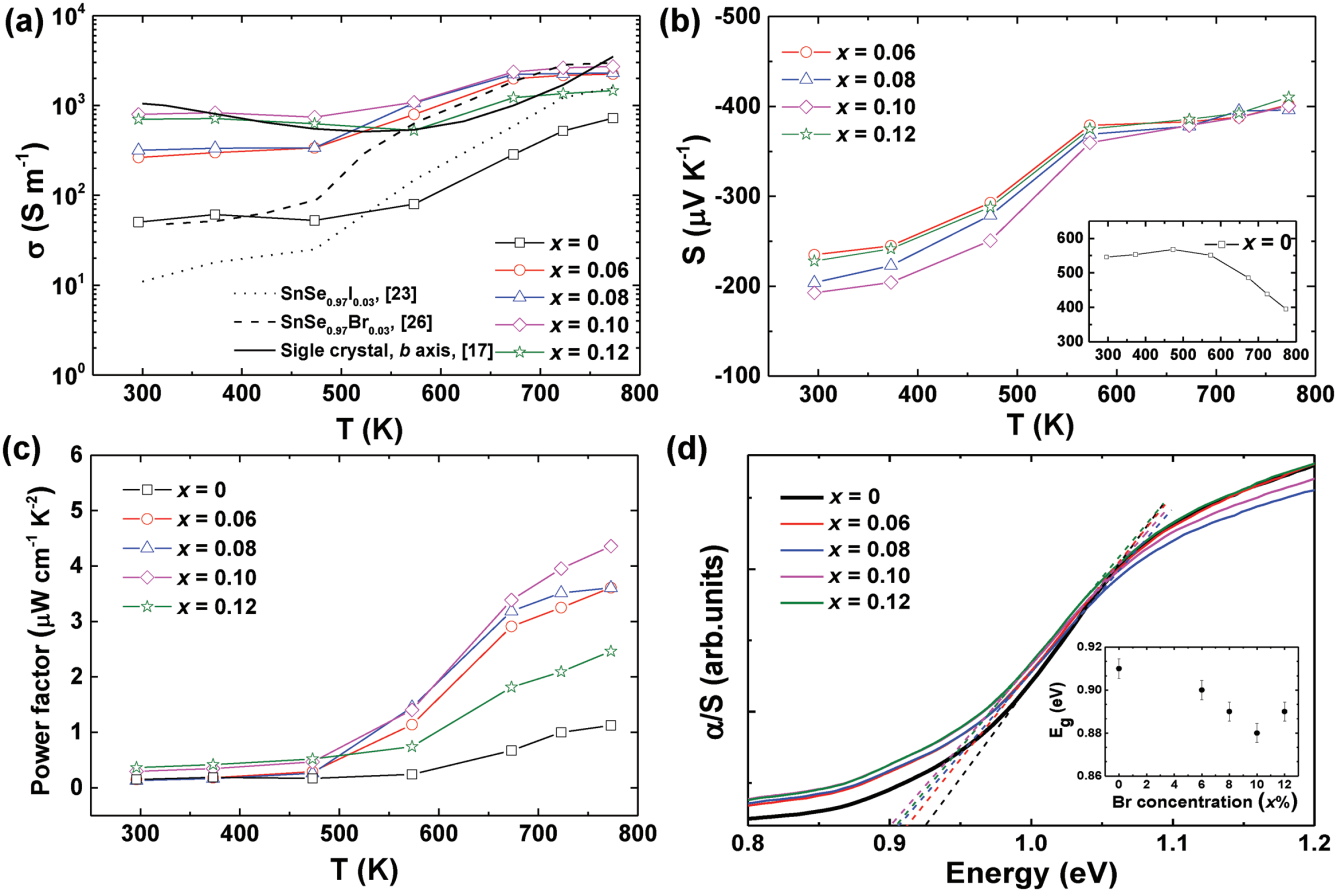
Temperature dependence of a) electrical conductivity b) Seebeck coefficient, and c) power factor for SnSe_1–_ *_x_*Br*_x_* (*x* = 0, 0.06, 0.08, 0.10, and 0.12). The electrical conductivity for SnSe_0.97_I_0.03_ 23 (dotted line), SnSe_0.97_Br_0.03_ 26 (dashed line), and single crystal SnSe^17^ (solid line) is included for comparison. The polycrystals were measured parallel to the hot pressing direction. The single crystal was measured along the *b* axis. d) Optical absorption spectra of SnSe_1–_ *_x_*Br*_x_* (*x* = 0, 0.06, 0.08, 0.10, and 0.12). The inset shows the calculated band gap of the samples.

**Figure 2:**
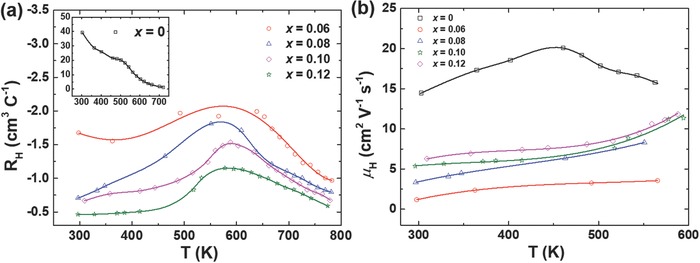
Temperature dependence of a) Hall coefficient and b) Hall mobility for SnSe_1–_ *_x_*Br*_x_* (*x* = 0, 0.06, 0.08, 0.10, and 0.12).

Since SnSe undergoes a structure transition from orthorhombic (*Pnma*) to orthorhombic (*Cmcm*) at about 780 K,^27^, ^28^ all the TE properties were measured below 780 K. **Figure** **1** shows the temperature dependence of a) electrical conductivity, b) Seebeck coefficient, and c) power factor for heavy Br‐doped SnSe_1–_ *_x_*Br*_x_* (*x* = 0, 0.06, 0.08, 0.1, and 0.12) measured parallel to the hot pressing direction. The electrical conductivity of SnSe_0.97_I_0.03_ 23 (dotted line), SnSe_0.97_Br_0.03_ 26 (dashed line), and single crystal SnSe^17^ (solid line) is included for comparison. The optical absorption spectra of all the samples were measured to verify the doping effect of Br (shown in Figure 1d). With increasing temperature, the electrical conductivity increased for undoped SnSe and all electron‐doped SnSe, showing typical semiconductor behavior. The room temperature electrical conductivity dramatically increased from ≈50 S m^−1^ in undoped SnSe to ≈800 S m^−1^ in Br‐doped SnSe_0.9_Br_0.1_, higher than those of I‐doped SnSe_0.97_I_0.03_ (≈10 S m^−1^),^23^ less Br‐doped SnSe_0.97_Br_0.03_ (≈50 S m^−1^),^26^ and even close to that of single crystal SnSe along the *b* axis (≈1000 S m^−1^).^17^ The Seebeck coefficient is positive for undoped SnSe, indicating a p‐type semiconductor. Br doping changed the conductive type from p to n in the whole temperature range, which is consistent with the Hall measurement as shown in **Figure** **2**a. Contributed from the enhancement in electrical conductivity, the SnSe_1–_ *_x_*Br*_x_* samples exhibited improvement in power factors, as shown in Figure 1c. The highest power factor is ≈4.5 µW cm^−1^ K^−2^ at 773 K for 10 atm% Br‐doped SnSe, which is four times higher than that of the undoped SnSe (≈1.1 µW cm^−1^ K^−2^). With increasing content of Br, the calculated band gap decreased from 0.91 eV for undoped SnSe to 0.88 eV for 10 atm% Br‐doped SnSe, further suggesting the doping of Br (see Figure 1d).

To clearly demonstrate the electronic transport properties of undoped and Br‐doped polycrystalline SnSe, the room temperature Hall carrier concentration and Hall carrier mobility together with real composition and density of all the samples are listed in **Table** **1**. The relative density of all the samples is higher than 90% although the evaporation of Sn and Se during melting and hot pressing. With increasing content of Br, the room temperature Hall electron concentration increased to ≈1.3 × 10^19^ cm^−3^, higher than those of I‐doped (higher solubility of Br in SnSe than I in SnSe) and less Br‐doped SnSe, suggesting the successful doping of Br. The carrier mobility decreased when Br was doped. Compared with the carrier mobility ≈250 cm^2^ V^−1^ s^−1^ of the single crystals along the *b* axis,^17^ the mobility of all the polycrystals is very low because of the scattering by the variety of defects in the crystal structure. Many factors, such as off‐stoichiometry, density (amount of holes), and microstructural features, etc. all together affect the carrier mobility, making the change of the carrier mobility for Br‐doped SnSe complex. But with the increased carrier concentration, the electrical conductivity of heavy Br‐doped SnSe is comparable to that of single crystal SnSe (see Figure 1a). We also investigated the temperature dependence of a) Hall coefficient and b) Hall mobility for SnSe_1–_ *_x_*Br*_x_* (*x* = 0, 0.06, 0.08, 0.10, and 0.12) and presented in Figure 2. With increasing content of Br, the absolute value of Hall coefficient decreased in the whole temperature range. The bipolar temperature increased after Br doping. The temperature dependence of Hall mobility (Figure 2b) shows an ionization impurity scattering at lower temperature for both undoped (*T* < 460 K) and heavy Br‐doped SnSe (*T* < 600 K).

**Table 1:** Room temperature real composition, density, Hall carrier concentration (*n*_H_), and carrier mobility (*µ*_H_) for SnSe_1–_ *_x_*Br*_x_* samples measured along the hot pressing direction

Nominal Comp.	Real Comp.	Density [g cm^−3^]	*n*_H_ [10^18^ cm^−3^]	*µ*_H_ [cm^2^ V^−1^ s^−1^]
SnSe	SnSe_1.03_	5.76	0.16	14.5
SnSe_0.94_Br_0.06_	SnSe_0.96_Br_0.05_	5.62	7.2	1.2
SnSe_0.92_Br_0.08_	SnSe_0.91_Br_0.07_	5.63	8.8	3.4
SnSe_0.90_Br_0.10_	SnSe_0.87_Br_0.073_	5.70	9.3	6.2
SnSe_0.88_Br_0.12_	SnSe_0.82_Br_0.08_	5.57	13.4	5.4
SnSe_0.97_I_0.03_ 23	SnSe_0.88_I_0.03_	5.81	0.046	–
SnSe_0.97_Br_0.03_ 26	–	5.80	1.8	0.6
SnSe (the *b* axis)^17^	–	–	0.3	250

**Figure 3:**
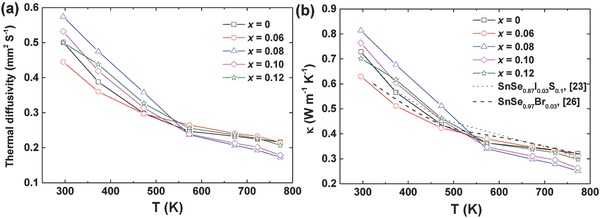
Temperature dependence of a) thermal diffusivity and b) total thermal conductivity for SnSe_1–_ *_x_*Br*_x_* (*x* = 0, 0.06, 0.08, 0.1, and 0.12) measured parallel to the hot pressing direction compared with reported data on SnSe_0.8_I_0.03_S_0.1_ 23 (dotted line) and SnSe_0.97_Br_0.03_ 26 (dashed line).

**Figure** **3** presents the temperature dependence of a) the thermal diffusivity, and b) total thermal conductivity of heavy Br‐doped SnSe_1–_ *_x_*Br*_x_* (*x* = 0, 0.06, 0.08, 0.1, and 0.12) in comparison with the reported data on n‐type SnSe_0.8_I_0.03_S_0.1_ 23 (dotted line) and less Br‐doped SnSe_0.97_Br_0.03_ 26 (dash line) measured parallel to the hot pressing direction. In a conservative way, considering that Br has a little higher atomic mass than Se, *C*_p_ of SnSe was used for the calculation of the total thermal conductivity for all the SnSe_1–_ *_x_*Br*_x_* samples. It should be mentioned that the total thermal conductivity (κ) is the sum of the electronic thermal conductivity (κ_e_ = *LσT*, where *L* is the Lorenz number) and lattice thermal conductivity (κ_L_). The contribution of electronic thermal conductivity could be negligible due to the low electrical conductivity (<3000 S m^−1^) shown in Figure 1a. Therefore, the lattice thermal conductivity is almost the same as the total thermal conductivity. As the thermal conductivity depends on many factors such as density, off‐stoichiometry, and slightly different preferential orientation, etc., the room temperature thermal conductivity of Br‐doped samples looks random. With increasing temperature, the total thermal conductivity of all the samples decreased. The lowest room temperature thermal conductivity is ≈0.63 W m^−1^ K^−1^ for SnSe_0.94_Br_0.06_, lower than 0.72 W m^−1^ K^−1^ for SnSe_0.87_I_0.03_S_0.1_, and the lowest thermal conductivity is ≈0.25 W m^−1^ K^−1^ at 773 K for SnSe_0.92_Br_0.08_, lower than ≈0.30 W m^−1^ K^−1^ for SnSe_0.87_I_0.03_S_0.1_ and ≈0.32 W m^−1^ K^−1^ for SnSe_0.97_Br_0.03_. This low thermal conductivity (at 773 K) is comparable to the results for single crystals measured along the *a* axis (e.g., 0.23 W m^−1^ K^−1^ for undoped SnSe and 0.27 W m^−1^ K^−1^ for Na‐doped SnSe).^17^, ^29^ With increasing content of Br, the thermal conductivity at 773 K decreased. The suppression of the bipolar effect (Figure 2a) possibly contributes to the decreased bipolar thermal conductivity at thigh temperature. Certainly, the low thermal conductivity of Br‐doped samples is also related to the defect scattering (we will discuss below). When Br is higher than 10 atm%, the thermal conductivity increased, which is maybe due to the doping limit confirmed by *E*_g_ data. In our previous study, lower thermal conductivity was achieved in 10 atm% SnS alloyed SnSe_0.97_I_0.03_ due to increased point defects,^23^ while heavy concentration of Br substituting Se in SnSe shows better effect in reducing the thermal conductivity.

**Figure 4:**
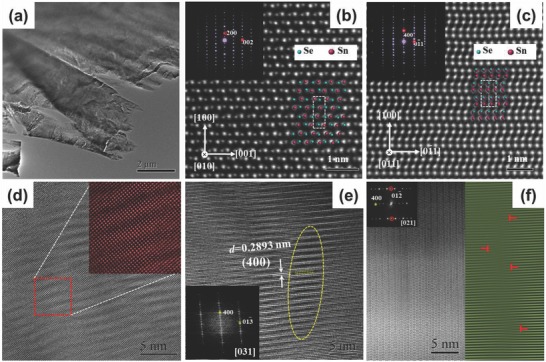
Microstructures of SnSe_0.9_Br_0.1_ sample. a) Low‐magnification TEM image shows the layered structure of the sample. HAADF‐STEM images of SnSe_0.9_Br_0.1_ viewed along b) [010] and c) [011]. The top‐left inset is the respective SAED pattern. The overlays show Se atoms in blue and Sn atoms in red. A single unit cell is marked by the dashed rectangle. d) A [100] HRTEM image with contrast fluctuations. e) HRTEM image shows the lattice distortion along the [100]* direction. The inset is corresponding FFTs. f) Many edge dislocations, and the extra half planes marked by ⊥ in magnified filtered image of the circular left area.

The intrinsically ultralow thermal conductivity of SnSe comes from the layered structure and strong anharmonicity of the chemical bonding.^30^ To further investigate the underlying mechanisms of the low thermal conductivity, we conducted a detailed microstructure investigation using high‐resolution transmission electron microscopy (HRTEM) and high‐angle annular‐dark‐field scanning transmission electron microscopy (HAADF‐STEM). The low‐magnification transmission electron microscopy (TEM) image in **Figure** **4**a shows a lamellar microstructure of SnSe_0.9_Br_0.1_ sample, which is consistent with layered crystal structure. Figure 4b,c shows HAADF‐STEM images viewed along [010] and [011] zone axes, respectively. The corresponding selected area electron diffraction (SAED) patterns inset in the upper left of the images. Since the contrast of HAADF‐STEM images is roughly proportional to Z^1.7^, where Z is the atomic number, the atomic column appeared bigger and brighter for Sn atoms (*Z* = 50) and smaller and darker for Se atoms (*Z* = 34) shown in Figure 4b. Along the [011] direction, the atomic columns of Sn and Se overlapped (see Figure 4c). Due to the similar atomic radii between Se and Br, a well integrality of lattice structure was achieved in spite of the substitution of Se with heavy content of Br, which is good for the electronic transport. The dashed rectangles represent the unit cell shown in Figure 4b,c. The two atom‐thick slabs marked by dashed rectangle were corrugated, forming a zigzag‐like Sn‐Se chains along the [001] direction. Some lattice distortions and defects were observed in the SnSe_0.9_Br_0.1_ sample. Figure 4d shows a [100] HRTEM image with contrast fluctuations. From the enlarged view inserted in the upper right, we can see the fluctuations clearly which is due to the composition fluctuations caused by Br doping. In Figure 4e, the spacing of the (400) planes in some areas as marked by the dashed line has a slight change. This lattice distortion is responsible for the intensity streaking along the [100]* direction in the corresponding fast Fourier transforms (FFTs) (see inset of Figure 4e). Figure 4f is a [021] image of SnSe_0.9_Br_0.1_, where the variety of images contrast induced by the dislocations is remarkable. In order to distinctly show the structure of the dislocation core, the filtering processing for Figure 4f was performed. From the filtered image shown in right part of Figure 4f, the inserted half planes parallel to (012) plane are clearly shown, which could significantly enhance the high frequency phonon scattering and contribute to the decreased high‐temperature thermal conductivity in Br‐doped SnSe samples.

**Figure 5:**
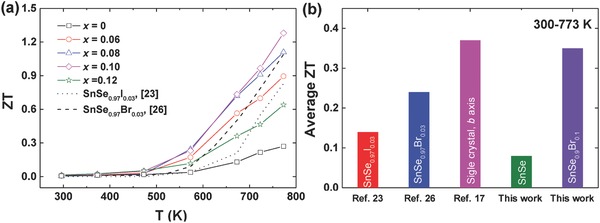
a) Temperature dependence of *ZT* for SnSe_1–_ *_x_*Br*_x_* (*x* = 0, 0.06, 0.08, 0.10, and 0.12) measured parallel to the hot pressing direction compared with reported data on SnSe_0.97_I_0.03_ 23 (dotted line) and SnSe_0.97_Br_0.03_ 26 (dashed line). b) Comparison of average *ZT* between 300 and 773 K for undoped SnSe (this work, green), 10 atm% Br‐doped SnSe (this work, purple), single crystal SnSe^17^ (the *b* axis, pink), SnSe_0.97_I_0.03_ 23 (red), and SnSe_0.97_Br_0.03_ 26 (blue).

**Figure** **5**a shows the temperature dependence of *ZT* for the SnSe_1–_ *_x_*Br*_x_* (*x* = 0, 0.06, 0.08, 0.10, and 0.12) samples. Due to the decreased thermal conductivity and increased electrical properties, the peak *ZT* increased with increasing Br content. The highest *ZT* value of ≈1.3 at 773 K was obtained in 10 atm% Br‐doped SnSe, which is significantly higher compared to the undoped SnSe (*ZT* ≈0.3 at 773 K) sample and also higher than the previously reported maximum *ZT* of I‐doped (*ZT* ≈0.8 at 773 K),^23^ and less Br‐doped (*ZT* ≈1.1 at 773 K) n‐type SnSe.^26^ To further clarify the anisotropy of SnSe, the TE properties perpendicular to the hot pressing direction were also measured and presented in Figure S3 in the Supporting Information. Due to the comparable power factor but higher thermal conductivity, the *ZT* value perpendicular to the hot pressing direction is much lower than that parallel to the hot pressing direction. We also present the average *ZT*s between 300 and 773 K for undoped SnSe and 10 atm% Br‐doped SnSe compared with reported optimum data of SnSe_0.97_I_0.03_,^23^ SnSe_0.97_Br_0.03_,^26^ and single crystal SnSe,^17^ shown in Figure 5b. The average *ZT* of 10 atm% Br‐doped SnSe (≈0.35) is relatively higher than undoped SnSe (≈0.07), even close to that of single crystal SnSe (≈0.37) along the *b* axis.^17^

## 3 Conclusions

In summary, we demonstrated an enhanced figure‐of‐merit in n‐type polycrystalline SnSe through a synergistic approach of optimizing the carrier optimization and effectively scattering the phonons by heavy bromine doping. With increasing doping concentration of Br, the room temperature Hall carrier concentration increased from 1.6 × 10^17^ cm^−3^ (p‐type) in undoped SnSe to 1.3 × 10^19^ cm^−3^ (n‐type) in SnSe_0.88_Br_0.12_, contributing to an increased electrical conductivity and power factor. The composition fluctuation and edge dislocations effectively scattered the short‐to‐medium wavelength phonons, resulting in the decreased thermal conductivity. With enhanced electrical properties and low thermal conductivity, a highest *ZT* value of ≈1.3 at 773 K was achieved in SnSe_0.9_Br_0.1_ along the hot pressing direction.

## 4 Experimental Section

*Synthesis*: Stoichiometric SnSe_1–_ *_x_*Br*_x_* (*x* = 0, 0.06, 0.08, 0.10, and 0.12) were prepared by melting, ball milling, and hot pressing. The raw materials (≈13 g, Sn shot (99.8%, Alfa Aesar), Se shot (99.999%, Australian Elements) and SnBr_2_ powder (99.2%, Alfa Aesar)) were sealed into the double evacuated quartz tubes and slowly heated up to 1193 K at a rate of 100 K h^−1^ and kept at that temperature for 6 h, then slowly cooled at the same rate to 873 K and kept for 50 h, finally slowly cooled to room temperature. The obtained ingots were cleaned and sealed into a stainless steel jar inside an argon filled glove box and ball milled by a high energy ball mill SPEX 8000D (SPEX SamplePrep) for 2 min. The powder was loaded into the graphite die and hot pressed at 873 K for 7 min using alternating current press (ac‐HP) under an axial pressure of 50 MPa, producing a cylinder‐shaped sample with ≈12.7 mm in diameter and ≈15 mm in height. All the samples were cut from two directions (parallel and perpendicular to the hot pressing direction) and measured along both directions.

*Characterizations*: X‐ray diffraction spectra analysis was conducted on a Rigaku D/max 2500 PC instrument with Cu K_α_ (λ = 1.5418 Å) radiation and a scanning rate of 8 ° min^−1^ from two directions of the anisotropic samples. No obvious second phase was found when *x* ≤ 0.12 (see Figure S1a,b in the Supporting Information). The microstructures were investigated by a scanning electron microscope (SEM, Hitachi S4700) and a spherical aberration‐corrected (C_s_‐corrected) electron microscope (JEM‐ARM200F). The chemical composition was analyzed by an energy‐dispersive X‐ray spectrometer attached to SEM. Room temperature optical diffuse reflectance spectra of the powders were detected on a UV‐3600 UV–vis–NIR spectrophotometer system. The band gaps were calculated using the Kubelka–Munk function. The Seebeck coefficient (*S*) and electrical resistivity (ρ) were simultaneously measured using a commercial system (CTA‐3) from room temperature to 773 K to avoid the effect of structural transition. Temperature‐dependent Hall coefficient (*R*_H_) was measured using the van der Pauw technique under a reversible magnetic field of 1.5 T. Hall carrier concentration (*n*_H_) and Hall mobility (*µ*_H_) were calculated via *n*_H_ = 1/(*eR*_H_) and *µ*_H_ = *R*_H_/ρ, respectively. The thermal conductivity (κ) was calculated by κ = *DαC_p_*, where *D* is volumetric density determined by the Archimedes method, α is thermal diffusivity measured using a laser flash technique (Netzsch LFA457), and *C*_p_ is specific heat capacity taken from previous data.^17^ Due to the anisotropy of SnSe, both the electrical and thermal transport properties were measured along and perpendicular to the hot pressing direction. The uncertainty for the electrical conductivity is 3%, the Seebeck coefficient 5%, the thermal conductivity 7% (comprising uncertainties of 4% for the thermal diffusivity, 5% for the specific heat, and 3% for the density), so the combined uncertainty for the power factor is 10% and that for *ZT* value is 12%. In order to increase the readability of the curves, error bars for TE data were not shown in the figures.

## Conflict of Interest

The authors declare no conflict of interest.

## Supporting information

Supplementary
