# Supplementary material for: Heavy Doping by Bromine to Improve the Thermoelectric Properties of n‐type Polycrystalline SnSe
Source: Adv Sci (Weinh). 2018 Jul 31;5(9):1800598. doi: 10.1002/advs.201800598 (PMC6145293; doi:10.1002/advs.201800598)
Supplement: Supplementary file 1 — Supplementary [file ADVS-5-1800598-s001.pdf]

Copyright WILEY-VCH Verlag GmbH & Co. KGaA, 69469 Weinheim, Germany, 2018.

ADVANCED SCIENCE
Open Access

Supporting Information

for *Adv. Sci.,* DOI: 10.1002/advs.201800598

Heavy Doping by Bromine to Improve the Thermoelectric Properties of n-type Polycrystalline SnSe

*Shan Li, Yumei Wang, Chen Chen, Xiaofang Li, Wenhua Xue, Xinyu Wang, Zongwei Zhang, Feng Cao, Jiehe Sui, Xingjun Liu, and Qian Zhang**

Supporting Information

Heavy Doping by Bromine to Improve the Thermoelectric Properties of n-type Polycrystalline SnSe

Shan Li[a], Yumei Wang[b], Chen Chen[a], Xiaofang Li[a], Wenhua Xue[b], Xinyu Wang[a], Zongwei Zhang[a], Feng Cao[c], Jiehe Sui[d], Xingjun Liu[a, d], Qian Zhang[a*]

[a]*Department of Materials Science and Engineering, Harbin Institute of Technology, Shenzhen, Guangdong 518055, P.R. China*

E-mail: zhangqf@hit.edu.cn

[b]*Beijing National Laboratory for Condensed Matter Physics, Institute of Physics, Chinese Academy of Science, Beijing 100190, China*

[c]*School of Science, Harbin Institute of Technology, Shenzhen, Guangdong 518055, P.R. China*

[d]*State Key Laboratory of Advanced Welding and Joining, Harbin Institute of Technology, Harbin 150001, China*

1. XRD patterns of Br-doped $SnSe_{1-x}Br_x$

XRD patterns of $SnSe_{1-x}Br_x$ (x = 0, 0.06, 0.08, 0.1, and 0.12) bulk samples taken in the plane parallel (a) and perpendicular (b) to the hot pressing direction were presented in figure S1. All the diffraction peaks were indexed to orthorhombic layered structure (*Pnma* space group (PDF #48-1224)) without impurity phase within the detection limit in spite of heavy Br doping. The strong difference in diffraction intensity between (111) and (400) planes indicates the anisotropy of the samples, which is consistent with the lamellar microstructure shown in figure S2. Due to the similar ionic radius of Se^{2-} (1.98 Å) and Br^{1-} (1.96 Å), there is no obvious change of the lattice parameters.

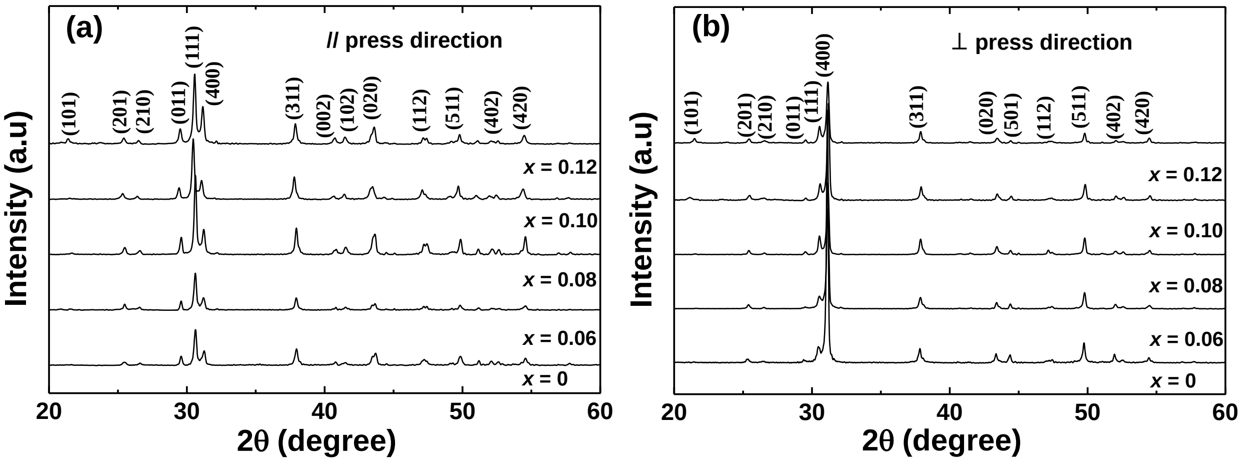

Figure S1. Room temperature XRD patterns for bulk samples $SnSe_{1-x}Br_x$ (x = 0, 0.06, 0.08, 0.10, and 0.12) taken in the plane (a) parallel to the hot pressing direction, and (b) perpendicular to the hot pressing direction.

2. Microstructure of Br-doped $SnSe_{0.9}Br_{0.1}$

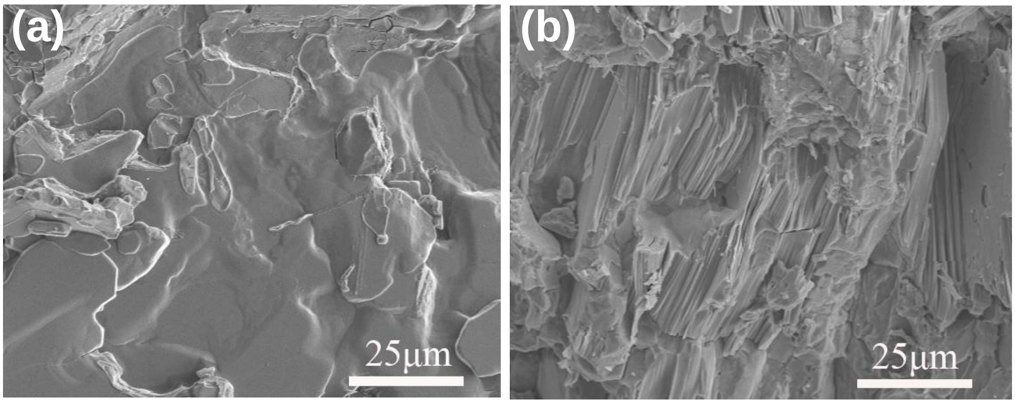

Figure S2. SEM images of the freshly fractured surface of Br-doped $SnSe_{0.9}Br_{0.1}$ bulk sample (a) parallel to the hot pressing direction and (b) perpendicular to the hot pressing direction.

3. TE properties for Br-doped $SnSe_{1-x}Br_x$ measured perpendicular to the hot pressing direction

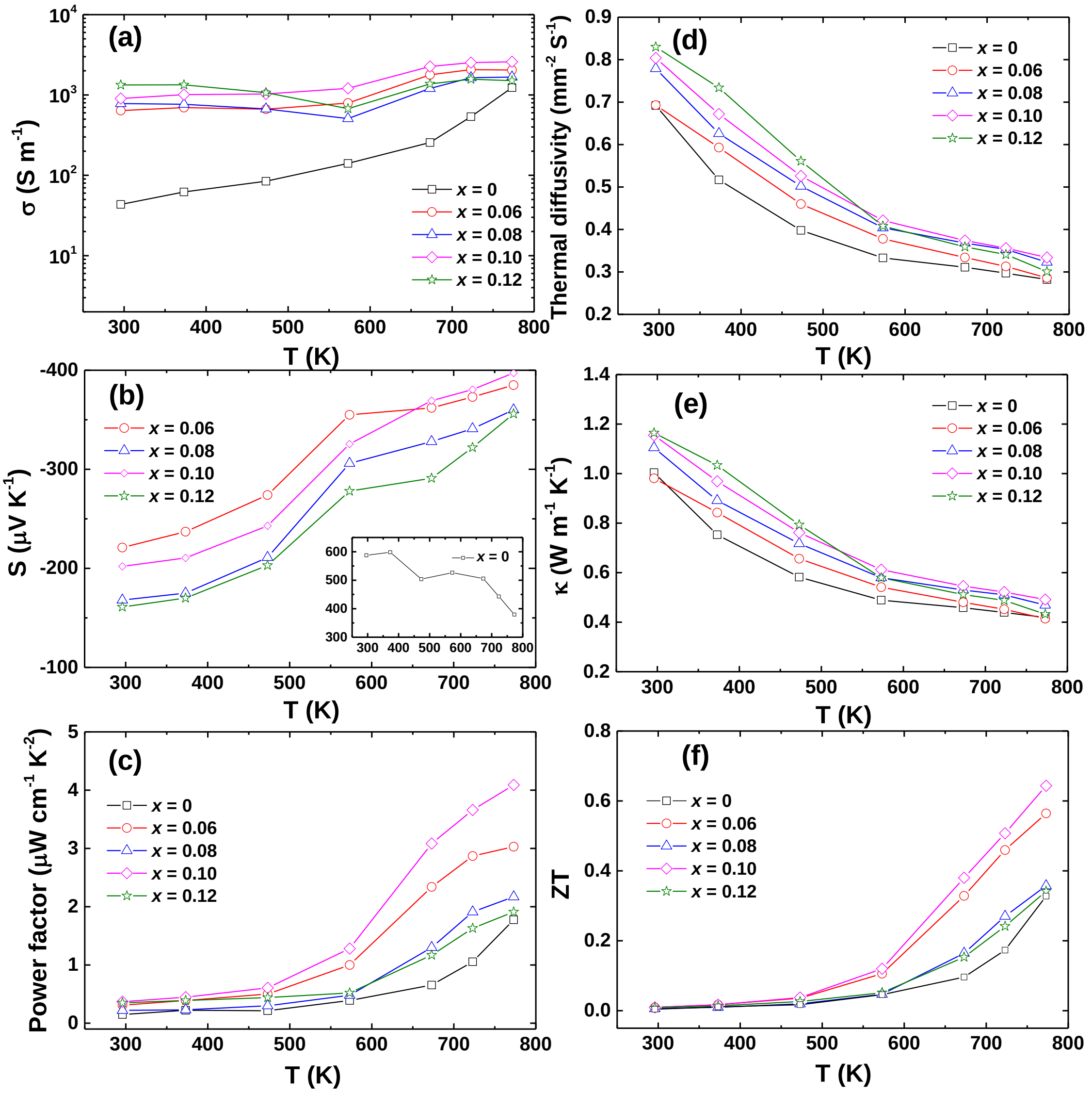

Figure S3. Temperature dependence of (a) electrical conductivity, (b) Seebeck coefficient, (c) power factor, (d) thermal diffusivity, (e) thermal conductivity, and (f) *ZT* for $SnSe_{1-x}Br_x$ (x = 0, 0.06, 0.08, 0.10, and 0.12) measured perpendicular to the hot pressing direction.
